# A cohort study of a tailored web intervention for preconception care

**DOI:** 10.1186/1472-6947-14-33

**Published:** 2014-04-15

**Authors:** Eleonora Agricola, Elisabetta Pandolfi, Michaela V Gonfiantini, Francesco Gesualdo, Mariateresa Romano, Emanuela Carloni, Pierpaolo Mastroiacovo, Alberto E Tozzi

**Affiliations:** 1 Bambino Gesù Children’s Hospital IRCCS, Epidemiology Unit, Piazza S. Onofrio 4, Rome 00165, Italy; 2 Department of Onco-Ematology and Transplantation Medicine, Bambino Gesù Children’s Hospital IRCCS, Piazza S. Onofrio 4, Rome 00165, Italy; 3 Alessandra Lisi International Centre on Birth Defects and Prematurity, Via Carlo Mirabello 14, Rome 00192, Italy

**Keywords:** Preconception care, Preconception health, Preconception counselling, Adverse pregnancy outcomes, Folic acid, Web intervention, Internet, Lifestyles

## Abstract

**Background:**

Preconception care may be an efficacious tool to reduce risk factors for adverse pregnancy outcomes that are associated with lifestyles and health status before pregnancy. We conducted a web-based cohort study in Italian women planning a pregnancy to assess whether a tailored web intervention may change knowledge and behaviours associated with risks for adverse pregnancy outcomes.

**Methods:**

The study was entirely conducted on the web on a cohort of Italian women of childbearing age. Data collected at baseline on health status, lifestyles and knowledge of risk factors for adverse pregnancy outcomes were used for generating a tailored document including recommendations for folic acid supplementation, obesity and underweight, smoking, alcohol consumption, vaccinations, chronic and genetic diseases, exposure to medications. Prevalence of risk factors and knowledge was assessed 6 months after the intervention. Logistic regression models were used to explore the factors associated with risk factors after the intervention.

**Results:**

Of the 508 enrolled women, 282 (55.5%) completed the study after 6 months since the delivery of tailored recommendations. At baseline, 48% of the participants took folic acid supplementation (95% CI 43.2; 51.9) and 69% consumed alcohol (95% CI 64.7; 72.9). At the follow up 71% of the participants had a preconception visit with a physician. Moreover we observed a decrease of alcohol consumption (−46.5% 95% CI −53.28; −38.75) and of the proportion of women not taking folic acid supplementation (−23.4% 95% CI −31.0; 15.36). We observed an improvement in knowledge of the information about the preconception behaviours to prevent adverse pregnancy outcomes (20.9% 95% CI 14.6%; 27.1%). Having a preconception visit during follow up was significally associated to an increase in folic acid supplementation (OR 2.53 95% CI 1.40; 4.60).

**Conclusions:**

Our results suggest that a tailored web intervention may improve general preconception health in women planning a pregnancy. A web preconception intervention may be integrated with classic preconception care by health professionals. Clinical trials should be conducted to confirm these findings.

## Background

Maternal lifestyle and health status are associated with the risk of adverse pregnancy outcomes (APOs) [1-7]. Preconception care can effectively inform women on how to identify and reduce risk factors for APOs through appropriate prevention [8]. Specifically, preconception interventions include smoking and alcohol cessation, achievement of a proper weight, folic acid supplementation, review and update of vaccinations, appropriate management and therapy of chronic diseases. In fact, some of these interventions have been shown to have effects on maternal health status, while the effectiveness in reducing the incidence of APOs still needs substantial evidence [9].

The Internet has achieved a pivotal role as a source of health information for physicians and for the general public [10,11], and may be an effective tool for delivering health care interventions to patients. A large number of web interventions aimed at improving lifestyles (smoking, alcohol consumption, diet, physical exercise) have been conducted, showing various levels of efficacy [12].

Web interventions offer several advantages as compared to traditional interventions because of high penetration of the Internet in the general population, wide enrolment potential through social media, interaction and personalization, time and costs savings, anonymity and privacy maintenance [13]. Although women widely use the Internet to get information on fertility and pregnancy [14], preconception counselling or interventions to reduce risk factors for APOs have rarely been delivered through the web [15,16].

We conducted a cohort study to explore the efficacy of an informative and tailored intervention in a population of Italian women of childbearing age planning a pregnancy. To this aim, we assessed their knowledge and prevalence of risk factors for APOs at baseline, and six months after delivering a set of tailored recommendations through the web.

## Methods

### Study design

We conducted a cohort study in Italian women of childbearing age who were planning a pregnancy within a year. Women were recruited on a web platform where they were profiled through a questionnaire on knowledge and behaviours. A summary of the study is provided (Additional file 1). Participants were then provided a downloadable document including recommendations for preventing risk factors for APOs based on their profile. A fictitious example of a tailored document is provided (Additional file 2). Details of the questionnaires and of the recommendations included in the tailored document are discussed in the following sections.

Prevalence of risk factors and knowledge was assessed again 6 months after the intervention. The study was approved by the Bambino Gesù Children’s Hospital ethical committee.

### Population, promotion of the study and enrolment

The study was conducted from September 2011 to May 2013. A convenience sample was included in the study through inclusion of women consecutively visiting the platform and requesting to be enrolled. Eligibility and exclusion criteria were reviewed through a questionnaire on the study web platform. Eligibility criteria included: 1) female gender; 2) age 18–45 years; 3) residence in Italy; 4) Italian language spoken; 5) plan of getting pregnant within the following year; 6) active email address and internet access; 7) online informed consent. Women with an on-going pregnancy were excluded from the study. Women not satisfying eligibility criteria were recommended to visit a web site on risk factors prevention during preconception period and pregnancy. The study was entirely conducted on the web and participants were never interviewed in person.

The study was promoted through Facebook and through articles explaining the project. The articles were freely published on 10 web sites dedicated to women’s health and family care. Facebook posts promoting the study were published twice a week for 2 months. Enrolment continued from September 2011 to November 2012, while the remaining six months, until May 2013, were dedicated to follow-up only. No paid advertisement was ever used and no incentives were offered for participation in the study.

### Web platform and intervention

Women meeting inclusion criteria were provided unique credentials (unique identifier, user name, and password) to access their personal account. Multiple enrolments by the same participant were prevented through manual review of multiple records with identical email address, IP address and responses to questionnaires.

Upon enrolment, women were asked to fill in a questionnaire on social and demographic data, personal and family medical history, lifestyles and knowledge of risk factors for APOs. The investigated risk factors included: obesity and underweight, smoking, alcohol consumption, genetic diseases and malformation, underlying diseases, exposure to medications, need for vaccination against rubella, varicella and hepatitis B. Folic acid supplementation was also investigated. Based on the information on the participant’s risk factors, a tailored set of recommendations was provided through a downloadable document created through an algorithm embedded in the website. The recommendation set was prepared following the guidelines provided by the American College of Obstetricians and Gynecologists (ACOG) [17]. The tailored document included a summary of personal information and a number of recommendations profiled on the participant’s risk factors. Each recommendation included no more than 2 text pages and included: a) general information on condition or exposure; b) type and frequency of associated adverse events concerning pregnancy; c) recommendations either on strategies to change behaviour or on medical interventions. Finally, the participants were invited to have a preconception visit with a health professional (her general practitioner, her obstetrician/gynaecologist) in order to have a complete assessment and counselling before getting pregnant. Participants were also invited to show the tailored document to the physician during the preconception visit (Additional file 2).

### Follow-up

Participants were reminded of the recommendation document through a monthly email. Six months after the enrolment, participants were invited by email to fill in a questionnaire including the same information on knowledge and behaviours recorded at enrolment. The questionnaire also investigated if the participant had a preconception visit after enrolment before getting pregnant. Enrolled women could withdraw from the study at any moment.

### Definitions and statistical analysis

Incomplete and inconsistent questionnaires were excluded from the analysis.

We described the population of participants through their socio-demographic, clinical, and behavioural characteristics. Consanguinity was defined as union between people that are related as first cousin or closer. Family history included genetic diseases, birth defects and malformations up to the first cousin relationship. Underlying diseases included type 1 diabetes, hypertensions, epilepsy, hypo- and hyperthyroidisms, hyperphenylalaninemia and asthma. Regarding drugs consumption, we asked for any current therapy, including over-the-counter medications. Drinking alcohol and smoking were defined as drinking any quantity of alcohol and smoking any quantity of cigarettes. An obstetrician/gynaecologist visit was defined as a routine check-up visit with an obstetrician or a gynaecologist, not necessarily including preconception counselling. We defined a woman as needing a rubella or hepatitis B vaccination if she had a) a negative serological test or no test ever performed and b) no specific immunization received. Need of varicella vaccination was defined as a) a negative serological test or no test ever performed and b) no specific immunization received and c) no recall of clinical disease. At follow up, preconception visit was defined as a visit with a health professional (her general practitioner, her obstetrician/gynaecologist) in order to have a complete assessment and counselling before getting pregnant.

We calculated the difference (percentage and 95% CI) between the prevalence of risk factors, and the level of knowledge of risk factors for APOs, before and six months after the delivery of tailored recommendations.

We studied the proportion of participant not taking folic acid supplementation and the prevalence of the following risk factors at enrolment: no visit with an obstetrician/gynaecologist in the last year, alcohol consumption, smoking, being underweight (BMI <18.5) or overweight (BMI ≥25), the need for vaccination against varicella, rubella, and hepatitis B.

We also studied the knowledge of the following items: folic acid supplementation, timing of preconception counselling, inheritability of malformations and genetic diseases, age at risk for Down syndrome, importance of maintaining a normal weight to prevent adverse pregnancy outcomes, need of testing susceptibility to infectious diseases before pregnancy, risk of vaccine preventable diseases which are harmful during pregnancy, underlying maternal diseases, smoking, medications and drinking alcohol on pregnancy outcomes.

Differences in proportions were assessed through the Chi square or Fisher exact test as appropriate. Differences in continuous variables were assessed through the Student t test.

Moreover, through logistic regression, we explored the effect of the following variables on risk factor prevalence after the intervention: parity, obstetrician or gynaecologist visit in the past 12 months, Pap smear test performed in the last 5 years; having initiated a pregnancy before the end of follow up; having had a preconception visit before the follow up questionnaire. We performed multiple logistic regression models in which current pregnancy was included as a single independent variable or as an interaction term with the other variables. We also adjusted the analysis for the following variables: age, geographical area of residence, education, employment, obstetrician or gynaecologist visit before enrolment, any previous miscarriage, family history of malformations, chronic or genetic diseases, underlying disease, current continuous use of any medication.

We used the STATA statistical package to perform the statistical analysis; 95% confidence intervals have been calculated according to Newcombe [18].

## Results

**Figure 1:**
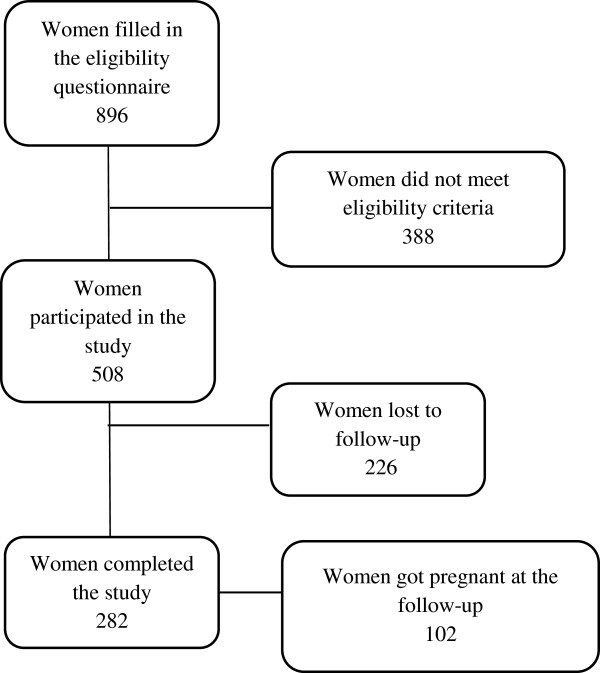
Flowchart of participants to the pilot study.

From September 2011 to November 2012, 896 women applied to participate in the study. 508 (56.7%) of them satisfied eligibility criteria and provided complete information at enrolment; 282 (55.5%) of them completed the follow-up (Figure 1).

Table 1 describes sociodemographic data, clinical data and prevalence of baseline risk factors among participants who completed the follow up and in those who did not. The majority of participants reported to be graduated, employed and planning their first pregnancy. Most participants had been visited by an obstetrician/gynaecologist in the past year and had undergone a Pap smear test in the past 5 years. Nearly 10% reported previous miscarriages and 12.5% had a family history of malformations, disabilities, genetic diseases or chronic diseases.

**Table 1:** Characteristics of participants and prevalence of risk factors for adverse pregnancy outcomes at baseline

	**Participants who completed the survey (n = 282)**	**Participants who did not complete the survey (n = 226)**	**Total (n = 508)**	***P***
**Age, yrs (SD)**	32.4 (4.6)	32.5 (5.1)	32.4 (4.8)	0.832
**University degree, N (%)**	184 (65.2)	122 (54.0)	306 (60.2)	0.012
**Employed, N (%)**	251 (89.0)	175 (77.4)	426 (83.9)	<0.001
**Residence**				
**North, N (%)**	125 (44.3)	88 (38.9)	213 (41.9)	0.061
**Center, N (%)**	114 (40.4)	85 (37.6)	199 (39.2)	
**South, N (%)**	43 (15.2)	53 (23.5)	96 (18.9)	
**Planning first pregnancy, N (%)**	213 (75.5)	164 (74.2)	377 (75.0)	0.734
**Previous miscarriage, N (%)**	16 (5.7)	33 (14.9)	49 (9.7)	0.001
**Obstetrician/Gynaecologist visit in the last year, N (%)**	243 (86.2)	183 (82.8)	426 (84.7)	0.298
**Pap-test in the last 5 years N (%)**	265 (94.0)	191 (86.4)	456 (90.7)	0.004
**Family history of malformation, N (%)**	31 (11.0)	32 (14.16)	63 (12.5)	0.257
**Consanguinity, N (%)**	5 (1.8)	9 (4.1)	14 (2.8)	0.12
**Underlying diseases, N (%)**	45 (16.0)	28 (12.8)	73 (14.6)	0.32
**Current medicine consumption, N (%)**	51 (18.1)	51 (22.9)	102 (20.2)	0.184
**No Folic acid supplementation N (%)**	148 (52.5)	118 (53.4)	266 (52.9)	0.839
**BMI <18.5 N (%)**	23 (8.2)	11 (4.9)	34 (6.7)	0.151
**BMI ≥ 25 N (%)**	52 (18.5)	55 (24.9)	107 (21.3)	0.083
**Need of vaccination against rubella N (%)**	55 (19.5)	52 (23.4)	107 (21.2)	0.285
**Need of vaccination against varicella N (%)**	37 (13.1)	30 (13.5)	67 (13.3)	0.913
**Need of vaccination against hepatitis b N (%)**	117 (41.5)	103 (46.6)	220 (43.7)	0.251
**Smoking (any quantity) N (%)**	37 (13.1)	48 (21.7)	85 (16.9)	0.011
**Drinking alcohol (any quantity) N (%)**	202 (71.6)	148 (67.0)	350 (69.6)	0.259

Missing values may affect denominators of different items.

More than a half of participants were not taking folic acid supplementation; nearly 70% reported to drink alcohol, while smoking was less prevalent (16.9%); one fifth was overweight; nearly 15% reported to have an underlying disease. One fifth reported to take medicines. Regarding immunization against preventable infectious diseases, 21.2%, 13.3%, and 43.7% needed a vaccination against rubella, varicella and hepatitis B respectively.

Women who did not complete the follow-up were less frequently graduated and employed, were more frequently overweight and more frequently reported previous miscarriages and smoking. Moreover, they had performed a Pap test less frequently compared to those who completed the follow-up.

Table 2 shows risk factors prevalence after the intervention and reports the differences between prevalence figures at the beginning and at the end of the study. The highest variation was observed for alcohol consumption (−46.45%; 95% CI −53.28; −38.75), followed by folic acid supplementation (−23.4%; 95% CI −31.0; 15.36). Susceptibility to hepatitis B (−22.34%; 95% CI −29.5; −14.84) and rubella (−13.83%; 95% CI −19.29; 8.46) also decreased significantly. Moreover, at follow up, 200/282 (70.9%) reported to have had a preconception visit before getting pregnant and 102/282 (36.2%) reported to have got pregnant.

**Table 2:** Comparison of prevalence of risk factors for APOs before and after the intervention

	**Prevalence of risk factors for APOs after intervention n = 282 (%)**	**Difference, % (95% CI)**	***P***
**No folic acid supplementation**	82 (29.1)	−23.4 (−31.0; 15.36)	<0.001
**BMI < 18.5**	16 (5.7)	−2.48 (−6.84; −1.79)	0.25
**BMI ≥ 25**	61 (21.6)	3.19 (−3.43; 9.78)	0.34
**Need of vaccination against rubella**	16 (5.7)	−13.83 (−19.29; 8.46)	<0.001
**Need of vaccination against varicella**	16 (5.7)	−7.45 (−12.38; 2.64)	0.002
**Need of vaccination against hepatitis b**	54 (19.1)	−22.34 (−29.5; −14.84)	<0.001
**Smoking, any quantity**	16 (5.7)	−7.45 (−12.38; 2.64)	0.002
**Drinking alcohol, any quantity**	71 (25.2)	−46.45 (−53.28; −38.75)	<0.001

Regarding participants’ knowledge of risk factors for APOs, the overall level of knowledge was high at baseline, with the exception of knowledge on adverse effects of overweight and obesity, teratogenicity of some drugs, alcohol consumption, maternal underlying diseases and infectious diseases (Table 3). In comparison with women who did not complete the follow up, the participant who ended the study had a higher knowledge of APOs associated to smoking (97.9% vs 93.9% p = 0.023) and of the effect of vaccinations to prevent harmful infection diseases (85.8% vs 75.7% p = 0.004) (Table 3).

**Table 3:** Knowledge of risk factors for adverse pregnancy outcomes at baseline

	**Participants giving a correct answer at baseline among those who completed the survey n = 282 (%)**	**Participants giving a correct answer at baseline among those who did not complete the survey n = 226 (%)**	**Total (n = 508)**	***P***
**General preconception behaviours**	199 (70.6)	146 (67.6)	345 (69.3)	0.476
**Folic acid supplementation**	270 (95.7)	201 (93.1)	471 (94.6)	0.189
**Timing of preconception counselling**	221 (78.4)	160 (74.8)	381 (76.8)	0.346
**Inheritability of malformations and genetic diseases**	233 (82.6)	171 (79.9)	404 (81.5)	0.441
**Age at risk for Down syndrome**	263 (93.3)	202 (94.4)	465 (93.8)	0.607
**Maintaining a normal weight to prevent adverse pregnancy outcomes**	276 (97.9)	210 (98.1)	486 (98.0)	0.839
**Overweight and obesity**	37 (13.1)	30 (14.0)	67 (13.5)	0.772
**Underlying maternal diseases**	55 (19.5)	35 (16.4)	90 (18.1)	0.368
**Smoking**	276 (97.9)	201 (93.9)	477 (96.2)	0.023
**Medications**	34 (12.1)	33 (49.3)	67 (13.5)	0.278
**Drinking alcohol**	164 (58.2)	128 (59.8)	292 (58.9)	0.71
**Need of testing susceptibility to infectious diseases**	242 (85.8)	162 (75.7)	404 (81.5)	0.004
**Immunization before pregnancy**	147 (52.1)	98 (45.8)	282 (56.9)	0.162

At the end of the study, the participants showed a significant increase in knowledge of correct preconception behaviours. Correct knowledge of timing of preconception counselling decreased with respect to baseline (−7.4%; 95% CI −14.5; 0.3) (Table 4).

**Table 4:** Comparison of knowledge of risk factors for APOs before and after the intervention

	**Participants giving a correct answer at follow up among those who completed the survey n = 282 (%)**	**Difference, % 95% CI**	***P***
**General preconception behaviours**	258 (91.5)	20.9	<0.001
		(14.6; 27.1)	
**Folic acid supplementation**	275 (97.5)	1.7	0.24
		(−1.3; 5.1)	
**Timing of preconception counselling**	200 (70.9)	−7.4	0.04
		(−14.5; 0.3)	
**Inheritability of malformations and genetic diseases**	128 (45.4)	−37.2	<0.001
		(−44.2; 29.6)	
**Age at risk for Down syndrome**	267 (94.7)	1.4	0.48
		(−2.6; 5.5)	
**Maintaining a normal weight to prevent adverse pregnancy outcomes**	275 (97.5)	−0.3	0.78
		(−3.1; 2.4)	
**Overweight and obesity**	33 (11.7)	−1.4	0.61
		(−6.9; 4.1)	
**Underlying maternal diseases**	42 (14.9)	−4.6	0.15
		(−10.8; 1.6)	
**Smoking**	274 (97.2)	−0.7	0.59
		(−3.6; 2.1)	
**Medications**	34 (12.1)	0	1,00
**Drinking alcohol**	173 (61.3)	3.2	0.44
		(−4.9; 11.2)	
**Need of testing susceptibility to infectious diseases**	251 (89.0)	3.2	0.25
		(−2.3; 8.7)	
**Immunization before pregnancy**	128 (46.0)	−6.74	0.11
		(−14.85; 1.5)	

The multivariate analysis (Table 5) showed that women who had a visit with an obstetrician/gynaecologist in the last 12 months were less frequently underweight (0.20 OR; 95% CI 0.06; 0.63) and less frequently drank alcohol (0.42 OR; 95% CI 0.18; 0.99) after the intervention; women who had a preconception visit at follow up were more frequently taking folic acid supplementation (2.53 OR; 95% CI 1.40; 4.60) and had more frequently a high BMI (2.18 OR; 95% CI 1.04; 4.58); women who had performed a Pap-test in the last 5 years were less frequently overweight (0.27 OR; 95% CI 0.08; 0.95) and women who had got pregnant during follow-up were more frequently taking folic acid supplementation (3.7 OR; 95% CI (1.96; 7.19), had more frequently a high BMI (2.25 OR; 95% CI (1.24; 4.09), were less frequently immunized against rubella (0.11 OR; 95% CI (0.01; 0.88) and less frequently drank alcohol (0.20 OR; 95% CI (0.10; 0.42) after the intervention.

**Table 5:** Factors associated with risk factors for adverse pregnancy outcomes

	**Previous pregnancies **	**Obstetrician or gynaecologist visit in the 12 months before enrolment**	**Pap smear test performed in the last 5 years before enrolment**	**Pregnancy at follow up OR**	**Preconception visit during follow up OR**
	**OR (95% CI) p**	**OR (95% CI) p**	**OR (95% CI) p**	**OR (95% CI) p**	**OR (95% CI) p**
**Folic acid supplementation**	1.02	1.49	1.42	**3.7**	**2.53**
	(0.528; 1.97)	(0.66; 3.369)	(0.442; 4.56)	**(1.96; 7.19)**	**(1.40; 4.60)**
	0.956	0.342	0.556	**0.000**	**0.002**
**BMI < 18.5**	1.052	**0.20**	2.89	0.99	0.71
	(0.31; 3.55)	**(0.06; 0.63)**	(0.31; 27.04)	(0.34; 2.95)	(0.24; 2.12)
	0.935	**0.006**	0.353	0.995	0.543)
**BMI ≥ 25**	1.34	1.81	**0.27**	**2.25**	**2.18**
	( 0.70; 2.61)	(0.63; 5.19)	**(0.08; 0.95)**	**(1.24; 4.09)**	**(1.04; 4.58)**
	0.388	0.271	**0.04**	**0.008**	**0.039**
**Need vaccination against rubella**	0.20	0.75	1.90	**0.11**	0.37
	(0.02; 1.64)	(0.16; 3.40)	(0.17; 21.57)	**(0.01; 0.88)**	(0.13; 1.09)
	0134	0.709	0.605	**0.037**	0.071
**Need vaccination against varicella**	1.66	1.80	Not given	0.33	1.10
	(0.53; 5.17)	(0.23; 14.37)		(0.09; 1.21)	(0.33; 3.649)
	0.380	0.578		0.095	0.869
**Need vaccination against hepatitis b**	1.14	1.26	0.72	0.80	1.47
	(0.54; 2.38)	(0.47; 3.39)	(0.20; 2.66)	(0.42; 1.53)	(0.71; 3.02)
	0.731	0.644	0.627	0.501	0.294
**Smoking**	0.431	2.18	0.31	0.14	0.49
	(0.09; 2.09)	(0.31; 2.10)	(0.04; 2.18)	(0.02; 1.10)	(0.16; 1.48)
	0.296	0.429	0.237	0.062	0.205
**Drinking alcohol**	1.04	**0.42**	2.13	**0.20**	1.44
	(0.52; 2.07)	**(0.18; 0.99)**	(0.58; 7.98)	**(0.10; 0.42)**	(0.73; 2.81)
	0.920	**0.05**	0.259	**0.000**	0.286

The bold numbers indicate the statistically significant values.

The other factors included in the analysis did not show a significant association with the outcomes nor interaction terms with current pregnancy showed a significant association.

## Discussion

A single, informative, personalized web intervention may decrease prevalence of risk factors for APOs in women planning a pregnancy.

We showed that constant monitoring of risk factors for APOs in women of childbearing age in order to inform public health actions and evaluate efficacy of interventions is feasible and easy. Our results also show that preventable risk factors for APOs are frequent in Italian women and deserve a thorough intervention through preconception counselling.

To our knowledge, the present intervention is the first web-based study to deliver preconception recommendations to a wide population of women of childbearing age planning a pregnancy. Moreover, this is one of the few studies aimed at improving general preconception health targeting multiple risk factors [6,7,9].

The Internet has been rarely used for preconception care promotion. A web-based study promoted folic acid supplementation among a selected population of women, resulting in an improved readiness to take folic acid in comparison with traditional educational methods [15]. A different study conducted through the web on a cohort of women of reproductive age investigated associations between preconception lifestyle and fertility, showing an effect of overweight and physical activity on fecundability [19-22]. Moreover, risk factors such as smoking, alcohol intake, obesity and physical inactivity reduce adherence to the preconception recommendation of folic acid intake [23]. Recently, an innovative computerized animated character has been developed and successfully used to identify and modify preconception risks in a pilot women community [16].

When analysing behavioural changes after the intervention, we observed a significant decrease in almost all the risk factors for APOs. A remarkable result is that 71% of women who completed the follow-up had a preconception visit with their physician. Actually, it may be possible that the observed reduction of risk factors could have been mainly driven by recommendations provided by physicians and not only by this intervention. Indeed, an increase in folic acid supplementation was significantly associated with having had a preconception visit during follow up.

Thirty-six per cent of women who completed the follow-up started a pregnancy within 6 months from enrolment. We observed that women that started a pregnancy during follow up more frequently started folic acid supplementation, received immunization against rubella, and quitted drinking alcohol. These results are in line with the observation that the motivation to change behaviour is stronger in women planning a pregnancy than in those of childbearing age [9].

We measured a high prevalence of risk factors at enrolment. Moreover, the women who did not complete the follow up had a higher prevalence of risk factors for APOs than those who completed the study. Interventions addressed at reducing the prevalence of multiple risk factors require strong engagement and time from the respondent [24]. As a matter of fact, more at-risk women received a longer set of recommendations, and were therefore asked a stronger effort to make a change. This entailed a higher risk of attrition. Prevention strategies should focus on women with higher risk profiles, and interventions should be appropriately designed for this high-risk group in order to effectively motivate them, thus preventing drop-outs.

Interestingly, we measured a high proportion of women needing a vaccination for one or more preventable infectious diseases that is in line with the Italian seroprevalence [25,26]. This result must be framed into the Italian immunization scenario. Rubella vaccine has been introduced in 1972; coverage for MMR vaccine only recently increased in children [27-29] and a special strategy for congenital rubella elimination has been recently implemented [30]. Varicella immunization is actively offered only in a few Italian Regions and the incidence of this disease is high in childhood. Immunization, however, is recommended in adolescents with no history of the disease [31]. Hepatitis B immunization is universally offered to infants in the first year of life since 1991 and a screening for hepatitis B is systematically performed during pregnancy.

Beside the likelihood of being captured by existing immunization strategies, we might have overestimated the proportion of women requiring a vaccination, as we included in the definition of need for vaccine also women who did not know their immunization status, and that therefore could actually have been already protected. However, it is likely that the proportion of women classified as “susceptible” according to our definition actually reflects the proportion of women that will be likely tested or vaccinated by their gynaecologist while planning a pregnancy.

Regarding knowledge of APOs, at enrolment most participants were already well informed on almost all items included in the study. This finding is apparently in contrast with the high prevalence of behaviours that pose a risk for pregnancy outcomes. A discrepancy between level of knowledge and actual behaviours has previously been reported [6,32]. After our intervention, knowledge of participants showed a significant change for the general information on preconception behaviours only.

Our study has a number of limitations. First of all, since all women received the intervention we cannot state that the changes observed are due to the intervention itself. In order to answer to this question, a properly designed trial would be needed.

Our web intervention was purely informative and aimed at delivering appropriate information on the risks associated with specific behaviours and medical conditions, as well as at promoting preconception counselling. The pivotal characteristics of our approach were: the possibility to fill in a questionnaire where and when the participant was more comfortable; a tailored set of recommendations that was immediately provided in a document which was always available for downloading; a summary of the participant’s clinical characteristics, included in the document front page, easy to show up to physicians during preconception counselling; a short and readable structured text illustrating the rationale, the actions, and the expected impacts on each woman for each recommendation; a positive reinforce for positive behaviours; a monthly reminder to resume the information provided at enrolment. Our study design does not include a control group, therefore we cannot draw sound conclusions on the efficacy of our intervention. Nevertheless, general knowledge on preconception behaviours increased consistently. It is likely that the potential confounders included in the analysis do not entirely explain the change in the risk factor prevalence, suggesting that the intervention itself may play a role. Nevertheless, our study shows that a web-based preconception intervention is feasible in women planning a pregnancy.

The validity of data collected through the web only may be questionable. However, other authors showed that web questionnaires are efficient means for data collection and we did not find evident inconsistencies during review of collected data [33].

As in other web-based interventions, one limitation is represented by the selection of the study participants.

The study was promoted on web-sites dedicated to women’ health and family care and through a Facebook account. The media used for the study promotion may have likely influenced the characteristics of the study population. The selected population may actively and frequently use the Internet for health information more. According to the National Institute of Statistics, in Italy 46.5% of women of childbearing age use the internet on this purpose [34]. Therefore the segment of population we have selected could be more motivated regarding participation in a preconception intervention study. The comparison with Italian Birth Registers [35] and with the Italian annual census [36] indicates that women enrolled in our study differed from the Italian population of women of childbearing age with respect to sociodemographic variables and lifestyles. In our sample, the mean age was 32.6. This data is comparable to the mean age (31.8 years) of Italian nulliparous women, according to the Italian Institute of Statistics [37]. No significant difference was found regarding smoking rate (16.9% vs. 16.9%) and BMI < 18.5 (8.2% vs. 9.6%) [36,38]. On the other hand, compared to Italian women of childbearing age, women included in our sample had a higher education level (60.2% vs. 20.7%), were more frequently employed (83.9% vs. 46.40%) and reported better medical conditions and lifestyle behaviours at baseline, in particular concerning overweight (21.3% vs. 37.0%), drug consumption (20.2% vs. 44.1%) and prevalence of chronic diseases (14.6% vs. 42.1%) [36]. Regarding miscarriages, our population presented a rate similar to that reported in the Italian Birth Registers (9.7% vs. 13.4%) [35]. These sample characteristics could have facilitated a more successful response to the intervention.

Nevertheless, although our results are not easily generalizable to the general population, our sample is likely representative of the specific population of women planning a pregnancy that frequently use the Internet for health purposes. Our results suggest that this specific population may benefit from a web-based intervention aimed at reducing preconception risk factor.

Furthermore, social engagement, active use of the Internet [39] and usability of the web page [40] may affect participation in web intervention studies.

We observed a remarkably high attrition that was, however, consistent with that reported by other studies [41]. Interestingly, unemployment, history of previous miscarriages and smoking positively predicted loss to follow-up. As discussed before, this finding suggests that a higher risk of attrition exists for women with a high-risk profile. Strategies for limiting loss to follow-up may include counsellor support, use of alternative means of communication as phone, updates of the intervention website, a more easy-to-read tailored document [40,42,43].

## Conclusions

Our study suggests that the web may be a valid tool for promoting preconception care. Web-based interventions have a low cost and may therefore represent a useful complement to traditional preconception counselling. Moreover, such interventions may be useful to continuously monitor the association between risk factors, preconception behaviours and pregnancy outcomes. A formal clinical trial with active engagement of participants and frequent recalls to prevent attrition will be useful to better address the effect of a web intervention to inform and implement prevention campaigns and health professionals’ activities. This study informs clinical trial studies regarding potential confounding variables to be included in the design.

## Abbreviations

ACOG: American College of Obstetricians and Gynecologists; APOs: Adverse pregnancy outcomes; BMI: Body mass index.

## Competing interest

The authors declare that they have no competing interests.

## Authors’ contribution

EA coordinated the study, designed the study and participated in the writing process and in the data review. EP designed the study, drafted the manuscript and participated in data review. MVG, FG, MR revised the final version of the manuscript. EC performed the statistical analysis. PM conceived the study, participated in its design, AET conceived the study, participated in its design and coordination and drafted the manuscript. All authors read and approved the final manuscript.

## Pre-publication history

The pre-publication history for this paper can be accessed here:

http://www.biomedcentral.com/1472-6947/14/33/prepub

## Supplementary Material

Additional file 1 Mammainforma box -Mammainforma project summary. Click here for file

Additional file 2 Tailored document - Fictitious example of Mammainforma tailored document, provided to participant after filling the questionnaire on knowledge and behaviours at the enrollment. Click here for file
