# Supplementary material for: A cohort study of a tailored web intervention for preconception care
Source: BMC Med Inform Decis Mak. 2014 Apr 15;14:33. doi: 10.1186/1472-6947-14-33 (PMC4021543; doi:10.1186/1472-6947-14-33)
Supplement: Additional file 1 — Mammainforma box -Mammainforma project summary. [file 1472-6947-14-33-S1.docx]

**Agrcola_Additional file 1 “Mammainforma” project**

“Mammainforma” is a web-based study dedicated to Italian women planning a pregnancy.

The aim of this study is to identify the participant’s risk factors for adverse pregnancy outcomes. In particular it looks at the effect of a web-based tailored preconception counseling intervention to improve general preconception health and knowledge.

**The study novelties**

The study offers a novel strategy for the prevention that is exclusively based on a web platform and is automatically tailored on the participant’s risk profile.

The present intervention is the first web-based study to deliver preconception and one of the few studies aimed at improving general preconception health targeting multiple risk factors

**Study promotion**

The study is promoted through Facebook and publication of free articles explaining the project on websites dedicated to women’s health and family care. The participation is free and the enrolled women are never interviewed in person.

**Participation**

Participant enrolled in the study is asked to fill in an online questionnaire to evaluate her risk factors for the adverse pregnancy outcomes. Once filled in, woman can download a tailored document providing recommendations to reduce the identified risk factors. Prevalence of risk factors and knowledge is assessed through a new questionnaire, six months after the intervention.
