# Supplementary material for: A cohort study of a tailored web intervention for preconception care
Source: BMC Med Inform Decis Mak. 2014 Apr 15;14:33. doi: 10.1186/1472-6947-14-33 (PMC4021543; doi:10.1186/1472-6947-14-33)
Supplement: Additional file 2 — Tailored document - Fictitious example of Mammainforma tailored document, provided to participant after filling the questionnaire on knowledge and behaviours at the enrollment. [file 1472-6947-14-33-S2.docx]

**Additional file 2**

Fictitious example of MammaInForma tailored document, provided to participant after filling the questionnaire on knowledge and behaviours at the enrollment.
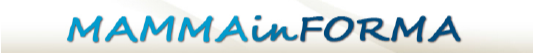


**Please, go to your health professional to have a preconception counselling and discuss this document with him/her.**

Preconception counselling aims at identifying and reducing your personal risk factors for adverse pregnancy outcomes, in order to improve your health status before starting a pregnancy.

During preconception counselling, your health professional will talk with you about your and you partner’s lifestyles, your family history and your medical history. When appropriate, he or she will suggest to you a set of interventions aimed at improving your and your partner’s health and at reducing your risk factors for adverse pregnancy outcomes before you get pregnant.

Your Personal profile

Dear Miss,

in the following section, you can find all the information you gave in the online questionnaire.

Please bring this document with you when you will have the preconception visit, as it will be useful to your health professional to better advice you.

| **Sociodemographic data** | |
| --- | --- |
| Age | 33 years old |
| Residence Region | Lazio |
| Education level | Graduated |
| Employment | Employed |
| **Health Status** | |
| Weight (Kg) | 55 |
| High (cm) | 165 |
| Body Mass Index (BMI) | 20.2 |
| Have you had your gynaecological visit during last year? | Yes |
| Have you performed a pap smear in the last five years? | Yes |
| Are you currently affected with:  High blood pressure? | No |
| Hyperphenylalaninemia/phenylketonuria? | No |
| Hypothyroidism? | No |
| Diabetes? | No |
| Epilepsy? | No |
| Asthma? | No |
| Hyperthyroidism? | No |
| Are you currently taking any medication? | No |
| **Family History** | |
| Are you and your partner related in any way? | No |
| Is anyone in your family affected by malformations, genetic diseases or chronic diseases? | No |
| **Infectious diseases** | |
| Have you ever had chickenpox? | Yes |
| Have you ever had a blood test to check if you are protected from: | |
| - Rubella? | Yes |
| - Chickenpox? | Yes |
| - Hepatitis B? | Yes |
|  |  |
| Have you ever had a vaccination for: | |
| - Rubella? | Yes |
| - Chickenpox? | Yes |
| - Hepatitis B? | Yes |
| **Lifestyles** | |
| Do you smoke? | No |
| Do you drink alcohol (even small quantities)? | Yes |
| Do you take folic acid supplementation every day? | No |
|  |  |
| **Previous pregnancies** | |
| Have you ever had more than one miscarriage? | No |
| Do you have children? | No |
| **Note:** |  |

**Folic Acid**

Folic acid is a vitamin of the B complex and you can get it through the food that contains it. At the beginning of pregnancy, the quantity of folic acid you can get by eating is not enough to prevent some birth defects, therefore it will be necessary to take a daily folic acid supplementation.

**Benefits of folic acid**

Folic acid is needed for synthesizing new cells, in particular blood cells and cells of the embryo. During the first days of pregnancy, embryo cells divide very rapidly, therefore increasing the need of folic acid.

Nowadays we know that the risk of birth defects is reduced if the woman gets right amounts of folic acid before getting pregnant and in the first three months of pregnancy.

**Folic acid reduces the risk of malformations**

During embryo development, neural cells form a structure similar to a tube, called ***neural tube***. It will generate the brain and the spinal cord, closing like a zip roughly 43 days after woman’s last period . When the neural tube does not completely close in its upper part, the embryo will not develop the brain. This condition is called ***anencephaly*** and is not compatible with life.

***Spina bifida*** is another severe condition that occurs when the neural tube remains open in its bottom part. Vertebras will not close properly and part of the spinal cord will poke out. After birth, the baby will have to undergo a surgical intervention. Nonetheless his/her legs will be paralyzed and the bladder will be not be able to efficiently pass urine. Often spina bifida is associated with ***hydrocephalus***, which consists in an excess of fluid in the brain cavities (ventricles).

Anencephaly and spina bifida occur in about 1/1000 pregnancies. In Italy, 550 cases are diagnosed every year.

**What you can do**

Start taking 0.4 mg of folic acid each day until the end of the third month of pregnancy.

**Alcohol**

According to the information collected in your questionnaire, you drink alcohol.

**Drinking alcohol can reduce fertility and seriously affect embryo development.**

Regardless of the amount of alcohol consumed, drinking alcohol can reduce fertility and seriously affect embryo development. Even a small amount of alcohol (i.e. a pint of beer or a glass of wine) can be dangerous, especially when consumed during the first weeks of pregnancy. Alcohol, in fact, can pass from the mother’s blood through the placenta through the umbilical cord. The foetus circulation will quickly reach the same alcohol level as that in the mother’s blood, and maintain it for a longer time.

**Alcohol effects on fertility and pregnancy**

Women who frequently drink alcohol can have fertility problems, such as amenorrhea (absence of menstruations), absence of ovulation and hormonal disorders resulting in a reduction or lack of fertility. Moreover, alcohol drinkers can frequently have adverse pregnancy outcomes, like miscarriage, premature birth, and newborn’s low birth weight. It is also well established that prenatal exposure to alcohol can determine foetal-alcoholic syndrome, characterized by facial anomalies and neurodevelopmental deficits.

Finally, alcohol consumption during pregnancy can cause problems that will become apparent as the child grows, such as learning and behavioural problems.

**What you can do**

Stop drinking alcohol and toast with no-alcoholic drinks before, during and after pregnancy!

Also remember that drinking alcohol is not safe for your baby while breastfeeding. A “safe” allowed alcohol amount is not known, but you can 100% avoid every single dangerous effect of alcohol intake if you stop drinking alcohol before, during and after pregnancy.

This is why many “no alcohol during pregnancy” campaigns are going on in different countries, and alcoholic beverages must have special labels. If you cannot control your alcohol desire, ask help to your doctor.

**GLOSSARY**

**Amenorrhea:** Amenorrhea means absence of [menstrual periods](http://www.medicinenet.com/script/main/art.asp?articlekey=92709), either on a permanent or temporary basis. Amenorrhea can be classified as primary or secondary. In primary amenorrhea, menstrual periods have never begun (by age 16). In secondary amenorrhea, menstrual periods are absent for three consecutive cycles or for more than six months, in a woman who was previously menstruating.

**Anencephaly:** Anencephaly is the absence of a major portion of the [brain](http://en.wikipedia.org/wiki/Brain), [skull](http://en.wikipedia.org/wiki/Skull), and [scalp](http://en.wikipedia.org/wiki/Scalp) that occurs during [embryonic development](http://en.wikipedia.org/wiki/Embryonic_development). This malformation is not compatible with life.

**Low birth weight:** weight at birth lower than 2.500 Kg.

**Phenylalanine:** it is an aminoacid present in the most common food proteins. Phenylketonuria is a disorder that occurs when the body is not able to use this aminoacid. It results in an excess of phenylalanine in the blood, urine and tissues leading to mental retardation.

**Hydrocephalus:** The term derives from Greek, meaning “water in the head”. It is characterized by an excessive accumulation of fluid in the brain.

**Foetal alcoholic syndrome:** a pattern of mental and physical defects that can develop in a foetus in association with high levels of alcohol consumption during pregnancy. Drinking alcohol during pregnancy can cause: craniofacial anomalies, central nervous system damage, growth retardation.

**Spina bifida:** [congenital disorder](http://en.wikipedia.org/wiki/Congenital_disorder) caused by the incomplete closing of the [embryonic](http://en.wikipedia.org/wiki/Embryo) [neural tube](http://en.wikipedia.org/wiki/Neural_tube). It can imply. If the opening is large enough, this allows a portion of the spinal cord to protrude through the opening in the bones, causing leg paralysis and bladder problems.

**Neural tube:** the [embryo](http://en.wikipedia.org/wiki/Embryo)'s precursor to the [central nervous system](http://en.wikipedia.org/wiki/Central_nervous_system), which includes the [brain](http://en.wikipedia.org/wiki/Brain) and he [spinal cord](http://en.wikipedia.org/wiki/Spinal_cord). If the neural tube does not close correctly during the first weeks of pregnancy, the foetus develops severe malformations, like anencephaly and spina bifida.
